# Changes in vessel density of the patients with narrow antenior chamber after an acute intraocular pressure elevation observed by OCT angiography

**DOI:** 10.1186/s12886-019-1146-6

**Published:** 2019-06-21

**Authors:** Zi-wei Ma, Wen-han Qiu, Dan-ni Zhou, Wei-hua Yang, Xue-feng Pan, Hong Chen

**Affiliations:** Department of Ophthalmology, The First People’s Hospital of Huzhou, Huzhou, 313000 Zhejiang Province China

**Keywords:** Intraocular pressure, Optical coherence tomography angiography, Vessel density

## Abstract

**Background:**

Although the pathogenesis of glaucoma is not fully understood,an elevated intraocular pressure (IOP) is a major factor contributing to its development and progression. The aim of this study was to investigate the changes in the vessel densities of the macula and optic nerve head (ONH) after an acute elevation in the intraocular pressure (IOP) observed using optical coherence tomography angiography (OCTA).

**Methods:**

This was a prospective comparative study of subjects with narrow anterior chamber angles who underwent laser peripheral iridotomies (LPIs). The IOP was measured before and one hour after the LPI. The retinal vessel densities of the macula and ONH were measured using OCTA at the baseline and one hour after the LPI.

**Results:**

A total of 64 eyes of 51 individuals were enrolled in this study, and 58 eyes of 43 individuals finally completed the study with a mean IOP rise of 10.5 ± 7.6 mmHg after the LPI. Based on the magnitude of the rise in the IOP, we divided the subjects into three groups: group A = IOP rise ≤10 mmHg, group B = 10 mmHg < IOP rise ≤20 mmHg, and group C = IOP rise > 20 mmHg. The vessel density did not differ after the acute IOP elevation in either the macular region or papillary region in group A or group B (*p* > 0.05), but there was a significant difference in group C (*p* < 0.05). However, when the subjects were not separated into groups, the vessel densities of the ONH and macular region did not differ between the measurements obtained at the baseline and one hour after the LPI (*p* > 0.05). The correlation existed in peripapillary and macular vessel density (*p* < 0.05).

**Conclusion:**

In these subjects with narrow antenior chamber, an acute mild or moderate IOP elevation for one hour after the LPI did not affect the vessel density in the macula or ONH, as examined using OCTA. However, when the IOP rise was greater than 20 mmHg, the macular and papillary vessel density decreased significantly.

## Background

Glaucoma is the second leading cause of blindness worldwide. It affected 64.3 million people in 2013, and this number is predicted to increase to 76 million by 2020 and 118 million by 2040 [1, 2]. Although the pathogenesis of glaucoma is not fully understood, an elevated intraocular pressure (IOP) and damage to the ocular blood flow are two of the major factors contributing to its development and progression [3–5]. Thus far, decreasing the IOP is the only consistent evidence-based treatment for glaucoma. The blood perfusions of the optic nerve head (ONH) and retina depend on the difference between the local arterial blood pressure and the IOP. However, there are complex regulatory mechanisms involved in the ONH and retinal microcirculation [6]. Relevant studies have proven that a change in the ocular perfusion pressure does not directly translate into a change in the blood flow in the capillary network of the ONH; however, the ONH microcirculation does have autoregulatory abilities. That is, it can maintain constant blood flow in the ONH during ocular perfusion pressure changes within a certain range [7, 8]. However, an elevated IOP may be the cause of decreased autoregulation [7].

Some of the techniques that have been used previously to detect the retinal blood flow include fluorescein angiography, indocyanine green angiography, laser Doppler flowmetry, color Doppler imaging, color Doppler optical coherence tomography (OCT), magnetic resonance imaging, and retinal photographic oximetry [9–15]. However, all of these methods have disadvantages associated with them, such as invasiveness, insufficient resolution, a large variation rate, and an inability to show capillaries. OCT angiography (OCTA) is a new noninvasive blood flow imaging technique that is based on split-spectrum amplitude-decorrelation angiography (SSADA) with OCT while acquiring retinal vascular imaging in only a few seconds. It allows one to measure the macula and ONH microcirculation in various retinal layers [16–20]. The stability and repeatability of using OCTA to measure the retinal blood flow have been widely demonstrated [21–23].

In this study, we used OCTA to assess whether there were changes in the retinal and papillary vessel densities in patients with an acute IOP rise after undergoing a laser peripheral iridotomy (LPI).

## Methods

### Participants

The study subjects included all of the patients with narrow anterior chamber angles who routinely and consecutively underwent LPIs and exhibited IOP elevations afterwards, from September 2018 to December 2018, at the First People’s Hospital of Huzhou, Zhejiang Province, China. The inclusion criteria were as follows: > 18 years old, narrow anterior chamber angles without occlusion, IOP ≤ 21 mmHg before the LPI, transparent refractive media, no medication, no retinal diseases, no optic nerve diseases, no diabetes, no ocular trauma, and OCTA images of sufficient quality (a signal strength index higher than 50 and accurate retinal stratification).

### Methods of operation and examination

This was a prospective comparative study of subjects with narrow anterior chamber angles who underwent LPIs. The device used to perform the LPIs was an Nd:YAG laser (Ellex Medical, Adelaide, South Australia) with the following parameters: wavelength = 1064 nm, energy = 6–12 MJ, and spot diameter = 30 um. Preoperatively, proparacaine hydrochloride eye drops were given once for the topical anesthesia, and an Abraham contact lens was placed in the conjunctival sac. An upper temporal or upper nasal iris treatment site was selected, the single pulse mode was used, and the slit lamp brightness was increased to shrink the pupil. After the LPI, tobramycin and dexamethasone eye drops were applied three times (once every 10 min), and then four times a day for 1 week.

Before the LPI and one hour after the LPI, the IOP was measured using noncontact tonometry (CT-60; Topcon Ltd., Tokyo, Japan). All of the IOP measurements were taken three times, and the mean value of the three measurements was used for the statistical analysis. Shortly after measuring the IOP, the vasculature of the macula and ONH was visualized using OCTA. If the IOP was greater than 30 mmHg, it was decreased with medication immediately following the examination. We used the RTVue XR OCT system (ReVue software, version 2017.1.1.155; Optovue Inc., Fremont, CA, USA) with the Angio Retina mode (6 × 6 mm) and the Angio Disc (4.5 × 4.5 mm) mode.

**Fig. 1:**
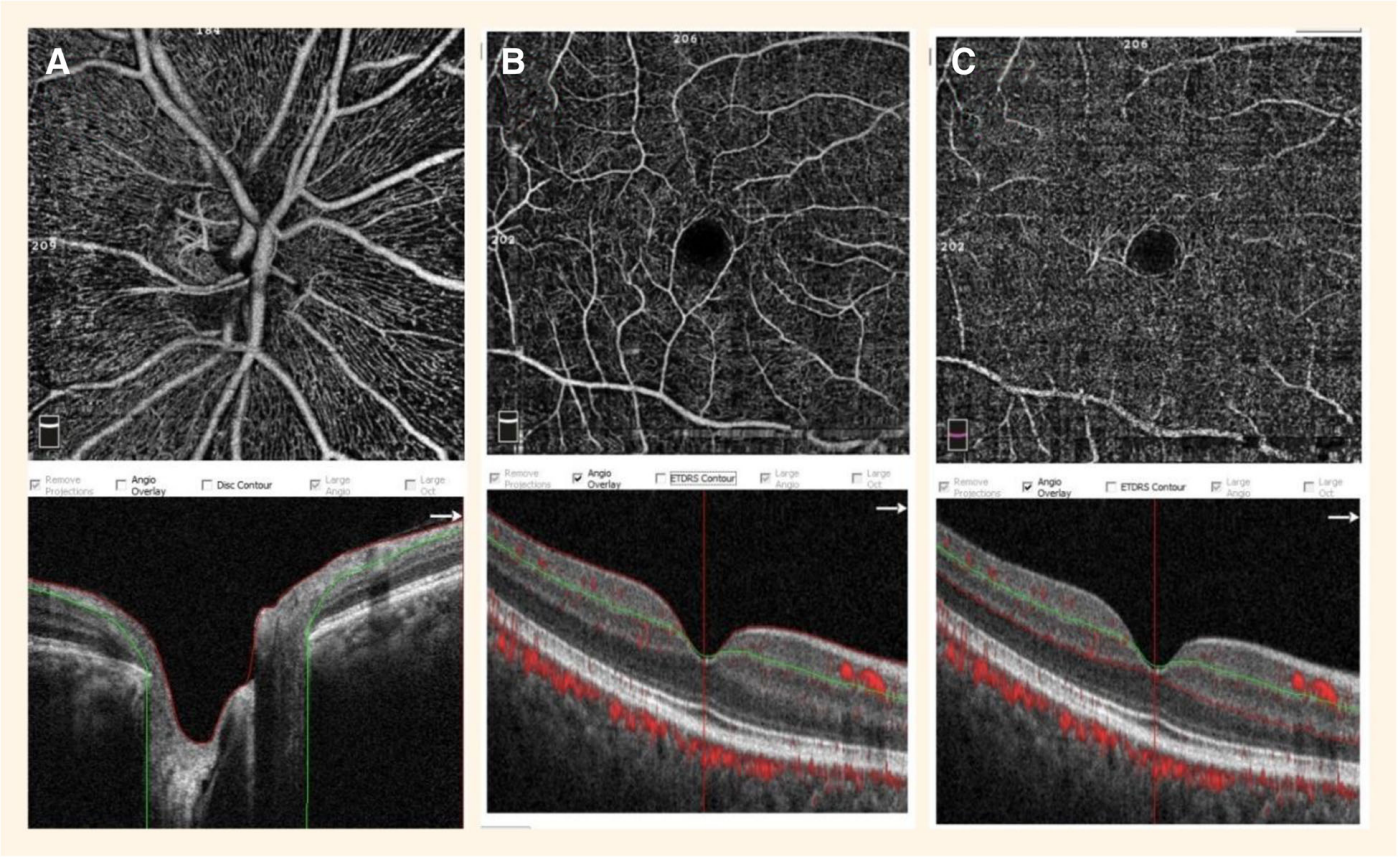
Optical coherence tomography angiography image of the optic nerve head region and macular region with capillary density measurements. **a** The vessel density (VD) measured in the papillary region. The picture identifies the radial peripapillary capillary layer in the papillary region (4.5 × 4.5 mm area), which extends from the upper boundary of the inner limiting membrane to the lower boundary of the nerve fiber layer. **b** The VD measured in the superficial retinal layer of the macular region (6 × 6 mm area), which extends from approximately 3 μm below the inner limiting membrane to 15 μm below the inner plexiform layer. **c** The VD measured in the deep retinal layer of the macular region (6 × 6 mm area), which extends from 15 μm below the inner plexiform layer to 70 μm below the inner plexiform layer

The details of the OCTA techniques have been described previously [24]. In order to evaluate the blood flow in the papillary region, we measured the vessel density (VD) in the radial peripapillary capillary (RPC) layer, which reaches from the upper boundary of the inner limiting membrane to the lower boundary of the nerve fiber layer. The ONH VD included the VD of the whole optic papilla (WVD), the VD inside the disc (IVD), and the peripapillary VD (PVD). To evaluate the blood flow in the macular region, we measured the VD of the superficial retinal layer, which extended from approximately 3 μm below the inner limiting membrane to 15 μm below the inner plexiform layer, and the deep retinal layer, which extended from 15 μm below the inner plexiform layer to 70 μm below the inner plexiform layer (Fig. 1).

## Statistical analysis

The statistical analysis was performed using the Statistical Package for the Social Sciences (version 17; SPSS Inc., Chicago, IL, USA). The measurements taken before and after the LPI were compared using the paired Student t test. Pearson correlation analysis was used to analyze the correlation between PVD and the vessel density of the macular region (MVD). All of the measurements were described as the mean ± standard error. A *p* value of less than 0.05 was considered to be statistically significant.

## Results

A total of 64 eyes of 51 individuals were enrolled in our study, of which 8 eyes of 8 individuals were excluded due to offset deviations or poor imaging signals (signal strength index lower than 50). Finally, 58 eyes of 43 individuals completed our study, including 13 males (18 eyes) and 30 females (40 eyes), with a mean age of 58.9 ± 4.9 years old (range = 49–74 years). The IOP rose from 14.7 ± 4.5 mmHg to 25.2 ± 10.5 mmHg, with a mean rise of 10.5 ± 7.6 mmHg.

According to the magnitude of rise in the IOP, we divided the subjects into three groups: group A = IOP rise ≤10 mmHg, group B = 10 mmHg < IOP rise ≤20 mmHg, and group C = IOP rise > 20 mmHg. There were 29 eyes (20 individuals) in group A, with a mean IOP rise of 4.2 ± 1.4 mmHg, there were 18 eyes (12 individuals) in group B, with a mean IOP rise of 12.8 ± 2.9 mmHg, and there were 11 eyes (11 individuals) in group C, with a mean IOP rise of 23.4 ± 2.7 mmHg (Table 1). Except for the IOP,there was no difference in age and sex composition among the three groups.

**Table 1:** General information from the three patient groups (group A = IOP increase ≤10 mmHg, group B = 10 mmHg < IOP increase ≤20 mmHg, and group C = IOP increase > 20 mmHg)

	Group A	Group B	Group C
Number of cases	29 eyes	18 eyes	11 eyes
Gender	6 males, 14 females	4 males, 8 females	3 males, 8 females
Age	60.7 ± 5.3 years old	56.6 ± 4.3 years old	57.9 ± 3.1 years old
IOP increase	4.2 ± 1.4 mmHg	12.8 ± 2.9 mmHg	23.4 ± 2.7 mmHg

*IOP* intraocular pressure

The age and IOP values are given as the mean ± standard error

**Fig. 2:**
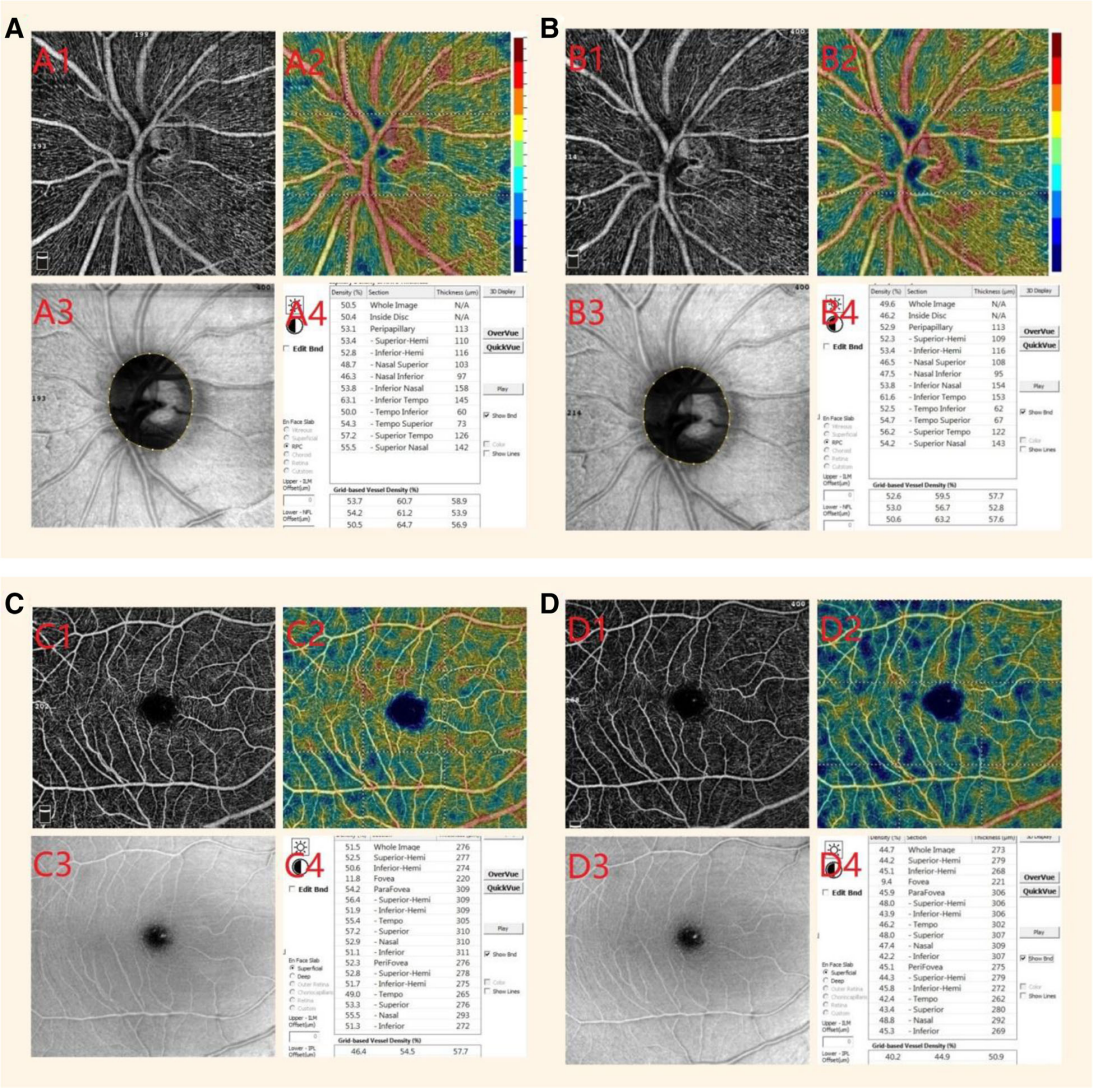
Images of the vessel densities measured before and one hour after the laser peripheral iridotomy. **a, b** Measurements of the papillary region: preoperative images (**a**1~**a**4) and postoperative images (**b**1~**b**4). **c, d** Measurements of the superficial retinal layer of the macular region: preoperative images (**c**1~**c**4) and postoperative images (**d**1~**d**4). Angioflow density images (**a**1**, b**1**, c**1**,** and **d**1), the corresponding color-coded angioflow density images (**a**2, **b**2, **c**2, and **d**2), the corresponding en face images (**a**3, **b**3, **c**3, and **d**3), and the corresponding data pictures (**a**4, **b**4, **c**4, and **d**4). The image quality and the position of the optic nerve head are similar in all of the corresponding A and B images, and the blood flow was reduced after an acute intraoperative pressure (IOP) elevation, especially inside the disc. The image quality and the position of the macula are similar in all of the corresponding (**c** and **d**) images, but the blood flow was reduced significantly after the acute IOP elevation

The VDs of the ONH region (containing the WVD, IVD, and PVD) measured at the baseline were not significantly different from the measurements obtained one hour after the LPIs in group A or group B in the RPC layer (WVD = 49.2 ± 4.5% versus 49.4 ± 4.4%, *p* = 0.152 and 49.6 ± 1.5% versus 49.8 ± 2.2%, *p* = 0.46, respectively; IVD = 46.8 ± 6.2% versus 47.3 ± 6.0%, *p* = 0.073 and 48.2 ± 4.9% versus 47.5 ± 3.0%, *p* = 0.275, respectively; PVD = 50.8 ± 5.4% versus 50.9 ± 5.4%, *p* = 0.973 and 52.4 ± 1.4% versus 52.5 ± 1.3%, *p* = 0.618, respectively). However, there were statistically significant differences in the group C values (WVD = 48.3 ± 2.6% versus 46.7 ± 3.4%, *p* = 0.013; IVD = 46.0 ± 3.2% versus 44.4 ± 3.6%, *p* = 0.012; PVD = 50.3 ± 3.3% versus 49.2 ± 4.3%, *p* = 0.012) (Table 2, Fig. 2).

**Table 2:** The vessel densities of the optic nerve head region at the baseline and one hour after the laser peripheral iridotomy (LPI) in the three patient groups (group A = IOP increase ≤10 mmHg, group B = 10 mmHg < IOP increase ≤20 mmHg, and group C = IOP increase > 20 mmHg)

	Group A		*p* value	Group B		*p* value	Group C		*p* value
	Baseline	After LPI		Baseline	After LPI		Baseline	After LPI	
WVD	49.2 ± 4.5	49.4 ± 4.4	0.152	49.6 ± 1.5	49.8 ± 2.2	0.464	48.3 ± 2.6	46.7 ± 3.4	0.013
IVD	46.8 ± 6.2	47.3 ± 6.0	0.073	48.2 ± 4.9	47.5 ± 3.0	0.275	46.0 ± 3.2	44.4 ± 3.6	0.012
PVD	50.8 ± 5.4	50.9 ± 5.4	0.973	52.4 ± 1.4	52.5 ± 1.3	0.618	50.3 ± 3.3	49.2 ± 4.3	0.012

*WVD* whole optic papilla vessel density, *IVD* vessel density inside the disc, *PVD* peripapillary vessel density

The values are given as the mean ± standard error %

There were no statistically significant differences between the VDs of the macular region (MVD) measured at the baseline and one hour after the LPI in group A or group B in either the superficial retinal layer or deep retinal layer (superficial layer MVD = 45.0 ± 4.4% versus 45.1 ± 4.5%, *p* = 0.889 and 49.3 ± 2.0% versus 49.2 ± 2.2%, *p* = 0.877, respectively; deep layer MVD = 50.2 ± 4.2% versus 50.3 ± 3.8%, *p* = 0.603 and 54.2 ± 3.0% versus 53.8 ± 2.5%, *p* = 0.137, respectively). However, there were significant differences between the values in group C (superficial layer MVD = 48.9 ± 2.2% versus 46.7 ± 2.9%, *p* = 0.020; deep layer MVD = 51.8 ± 2.8% versus 50.1 ± 3.0%, *p* = 0.041) (Table 3, Fig. 2).

**Table 3:** The vessel densities of the macular region (MVDs) at the baseline and one hour after the laser peripheral iridotomy (LPI) in the three patient groups (group A = IOP increase ≤10 mmHg, group B = 10 mmHg < IOP increase ≤20 mmHg, and group C = IOP increase > 20 mmHg)

	Group A		*p* value	Group B		*p* value	Group C		*p* value
	Baseline	After LPI		Baseline	After LPI		Baseline	After LPI	
MVDs	45.0 ± 4.4	45.1 ± 4.5	0.889	49.3 ± 2.0	49.2 ± 2.2	0.877	48.9 ± 2.2	46.7 ± 2.9	0.020
MVDd	50.2 ± 4.2	50.3 ± 3.8	0.603	54.2 ± 3.0	53.8 ± 2.5	0.137	51.8 ± 2.8	50.1 ± 3.0	0.041

*MVDs* superficial layer MVD, *MVDd* deep layer MVD

The values are given as the mean ± standard error %

Without dividing the subjects into groups, the VDs of the ONH and macular regions of all of the subjects did not differ significantly between the measurements obtained at the baseline and one hour after the LPI (WVD = 49.1 ± 3.5% versus 49.0 ± 3.8%, *p* = 0.508; IVD = 47.0 ± 5.3% versus 46.8 ± 4.9%, *p* = 0.435; PVD = 51.2 ± 4.2% versus 51.0 ± 4.4%, *p* = 0.160; superficial layer MVD = 47.1 ± 4.0% versus 46.7 ± 4.1%, *p* = 0.109; deep layer MVD = 51.8 ± 4.0% versus 51.4 ± 3.6%, *p* = 0.056) (Table 4).

**Table 4:** The vessel densities of the optic nerve head and macular regions at the baseline and one hour after the laser peripheral iridotomy (LPI) in all of the study subjects

	Baseline	After LPI	*p* value
WVD	49.1 ± 3.5	49.0 ± 3.8	0.508
IVD	47.0 ± 5.3	46.8 ± 4.9	0.435
PVD	51.2 ± 4.2	51.0 ± 4.4	0.160
MVDs	47.1 ± 4.0	46.7 ± 4.1	0.109
MVDd	51.8 ± 4.0	51.4 ± 3.6	0.056

*WVD* whole optic papilla vessel density, *IVD* vessel density inside the disc, *PVD* peripapillary vessel density, *MVDs* superficial layer macular vessel density, *MVDd* deep layer macular vessel density

The values are given as the mean ± standard error %

**Fig. 3:**
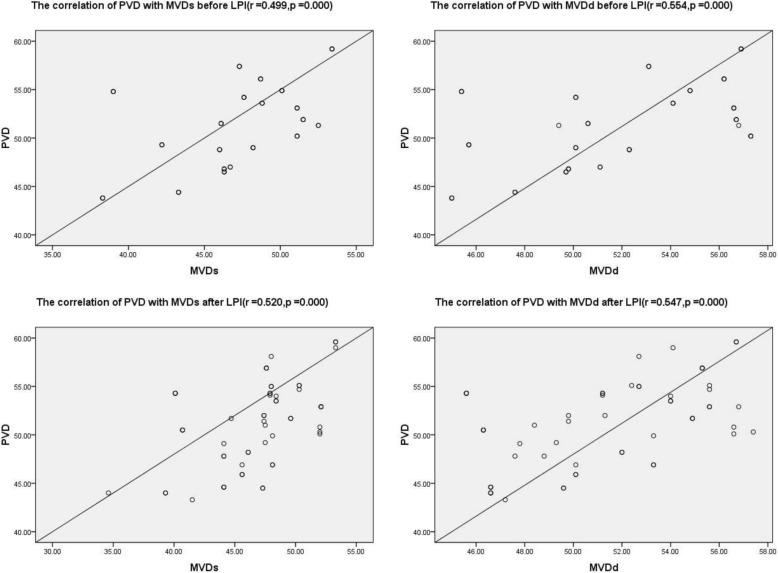
Images of the correlation of PVD with MVD (including MVDs and MVDd) before and after LPI. PVD: peripapillary vessel density, MVDs: superficial layer macular vessel density, MVDd: deep layer macular vessel density

The pearson’s correlation analysis showed that there was a positive correlation between PVD and MVD before and after LPI(r = 0.499,*p* = 0.000;r = 0.554,*p* = 0.000;r = 0.520,*p* = 0.000;r = 0.547,*p* = 0.000) (Fig. 3).

## Discussion

Currently, noninvasive diagnostic methods, such as OCTA, have become more and more popular, and they are gradually replacing invasive fundus examinations, like fluorescein angiography and indocyanine green angiography. OCTA takes advantage of the SSADA, which is based on the variations in the reflectance amplitude. It is sensitive to motion and flow in all directions, and it provides high quality three-dimensional angiography. Based on the results of our study, OCTA can provide a good indicator of the retinal vessel density in the optic nerve and macula, with a few exceptions, such as the consistency of the scan areas before and after the LPI, pupil size, and poor imaging signals. Occasionally a subject’s poor coordination could lead to inconsistencies in the scan areas before and after the LPI, so it is necessary to compare the two images immediately after the postoperative examination, and if necessary, conduct a reexamination or exclude the subject. In order to ensure good quality imaging, we used the slit lamp brightness to reduce the size of the pupils during the operation, instead of preoperatively shrinking the pupils with pilocarpine.

Recent studies using OCTA have demonstrated a reduced retinal blood flow in glaucoma cases when compared to control cases [25–27]. However, up until now, whether or not an elevated IOP has an effect on the retinal blood flow has not been determined, and there are not many related studies available in the literature. With the extensive developments that have been made in OCTA, we have a more intuitive and accurate understanding of the retinal blood flow, which is helpful for quantitative research. Animal experiments have shown that with a sharp increase in the IOP, the retinal blood flow decreases significantly [28, 29]. Hollo′ found that a large medical IOP reduction may result in a clinically significant increase in the peripapillary capillary perfusion in the retinal nerve fiber layer in young individuals with untreated high IOPs [30]. However, the research by Zhang et al. indicated that healthy eyes with acute and physiological IOP elevations of 10 mmHg or 15 mmHg for two hours did not show significant changes in the capillary VD in either the ONH or macula, as examined using OCTA [31]. The results of our study revealed that mild to moderate IOP increases have little effect on the macular and ONH VD, but an IOP elevation that exceeds 20 mmHg causes the vessel density to decrease significantly. The reason for this may be that the retinal autoregulation ability plays an important role, and when the IOP elevation exceeds the retinal autoregulation ability, the retinal blood flow is reduced, which is consistent with the results of the study by Cherecheanu et al. [7]

While we analyzed the correlation between PVD and MVD before and after LPI, it was interesting to find that there was a significant correlation, which suggested that the macular vessel density of glaucoma patients might also have vascular density damage. Some studies have shown that, glaucomatous damage to the macula often occurs early in glaucoma eyes [32, 33]. Although current studies assessed macula thickness change in glaucoma, only a few focused on the macula blood flow density, and most of them revealed a decrease vessel density in macula [34–36]. But Giacinto Triolo et al. [37] showed different results, and they found no significant difference in vessel perfusion density in the macular area between glaucoma and normal controls.

Previous IOP elevation models mainly employed prone provocative testing in a dark room [31, 38], while we employed an LPI to create a high IOP model, which was caused by the iris debris and inflammation after the LPI. As far as we know, the IOP after a prone provocative test in a dark room is time-sensitive, and its normalization begins to occur quickly; however, the high IOP model after an LPI can overcome these shortcomings. Nevertheless, the anterior chamber opacity may affect the OCTA signal after an LPI. The reason why we chose one hour after the LPI for the relevant examinations was because we observed another 29 patients after LPIs and found that the IOPs peaked about one hour after the LPIs, and that the anterior chamber transparency was acceptable. However, 8 eyes of 8 subjects were excluded due to the influence of the anterior chamber opacity, which led to offset deviations or poor imaging signals.

The limitations of this study are as follows. First, our study included only patients with narrow anterior chamber angles, and we do not know whether the results can be generalized to any other individuals. Second, in the papillary region, we only observed the RPCs, which made up a vascular network with the retinal nerve fiber layer around the optic disc, and they are considered to be vulnerable to retinopathies, such as retinal vascular occlusion and glaucoma. We did not observe the deep blood flow of the optic papilla [39, 40]. Third, the vessel density observed by OCTA is not exactly equal to blood flow,so blood flow and retinal perfusion may also be slightly compromised in groups A and B. However, OCTA may not be able to detect this. Fourth,the limited number of samples in this study, especially in the cases with IOP increases greater than 20 mmHg after the LPI, may have had an impact on the outcomes. Therefore, further research is necessary, including enlarging sample size, increasing observation indexes, and introducing other blood flow detection methods for comparison, etc.

## Conclusion

In conclusion, in these patients with narrow antenior chamber, an acute mild or moderate IOP elevation for one hour after an LPI did not affect the vessel density in either the macula or ONH, as examined using OCTA. However, when the IOP increase was more than 20 mmHg, the macular and papillary vessel density decreased significantly.

## Data Availability

The datasets used and analyzed during the current study are available from the corresponding author on reasonable request.

## Abbreviations

IOP: Intraocular pressure
IVD: The VD inside the disc
LPI: Laser peripheral iridotomy
MVD: The VD of the macular region
OCTA: Optical coherence tomography angiography
ONH: Optic nerve head
PVD: The peripapillary VD
RPC: Radial peripapillary capillary
SSADA: Split-spectrum amplitude-decorrelation angiography
VD: Vessel density
WVD: The VD of the whole optic papilla
